# Ventral and Dorsal Pathways Relate Differently to Visual Awareness of Body Postures under Continuous Flash Suppression

**DOI:** 10.1523/ENEURO.0285-17.2017

**Published:** 2018-02-13

**Authors:** Minye Zhan, Rainer Goebel, Beatrice de Gelder

**Affiliations:** 1 Faculty of Psychology and Neuroscience, Department of Cognitive Neuroscience, Maastricht University, Maastricht 6229EV, The Netherlands; 2 Department of Neuroimaging and Neuromodeling, Netherlands Institute for Neuroscience, Institute of the Royal Netherlands Academy of Arts and Sciences, Amsterdam 1105BA, The Netherlands; 3 Department of Computer Science, University College London, London WC1E 6BT, United Kingdom

**Keywords:** action perception, bodily expression, consciousness, continuous flash suppression, visual awareness

## Abstract

Visual perception includes ventral and dorsal stream processes. However, it is still unclear whether the former is predominantly related to conscious and the latter to nonconscious visual perception as argued in the literature. In this study upright and inverted body postures were rendered either visible or invisible under continuous flash suppression (CFS), while brain activity of human participants was measured with functional MRI (fMRI). Activity in the ventral body-sensitive areas was higher during visible conditions. In comparison, activity in the posterior part of the bilateral intraparietal sulcus (IPS) showed a significant interaction of stimulus orientation and visibility. Our results provide evidence that dorsal stream areas are less associated with visual awareness.

## Significance Statement

The occipital and parietal lobes of the human brain include two visual processing streams, a ventral one more involved in object recognition, and a dorsal one for spatial-, attention-, and action-related processes. It is currently still unclear what the relation is between activity in the dorsal processing stream and consciousness, as evidence so far has been scarce and inconsistent. Our study with whole-body stimuli shows that activity in the dorsal pathway is substantially less influenced by subjective stimulus awareness, while activity in the ventral pathway is significantly higher for consciously perceived stimuli. Our results clarify this important difference between visual perception in dorsal and ventral stream.

## Introduction

The occipito-temporal and parietal lobes of the human brain contain two major processing streams: the ventral stream is involved more in processes related to object recognition, and the dorsal one more in spatial processing, attention, and online control of actions (Milner and Goodale, 2006).

An important open question concerns the relation of these two processing streams to subjective awareness. A dissociation has been shown in patients with brain lesions: a patient with lateral occipital cortex damage could perform visually guided actions according to the size, shape or orientation of objects and tools, despite being unable to consciously differentiate those properties (Carey et al., 1996; Milner, 2012); patients with parieto-temporal cortex damage (McIntosh et al., 2004) or bilateral V1 damage (de Gelder et al., 2008) showed obstacle avoidance without being consciously aware of the obstacle. Addressing the relationship between the two streams in neurotypical participants requires controlled presentation of subjectively unseen stimuli, as can be achieved using the continuous flash suppression (CFS) method. Under CFS, the dichoptic presentation of a target stimulus and a dynamic noise pattern renders the target invisible for several seconds (Tsuchiya and Koch, 2005; Tsuchiya et al., 2006; Yang et al., 2014).

The two-stream view does not imply an absolute division, and processing of some object categories clearly involves both streams. For example, tools (Johnson-Frey, 2004; Culham et al., 2006) trigger activity related to the object category in ventral areas, but also to action-observation-execution in dorsal areas. Several functional MRI (fMRI) studies directly compared ventral and dorsal activity and their relationship with visual awareness using CFS. They varied in experimental designs, but all used stimuli of either tools only or together with faces. For the ventral stream, these studies consistently showed that the activity in ventral-lateral areas including the fusiform area and the lateral occipital area covaried with subjective perceptual awareness for both faces and tools, and that the activity for invisible faces/tools was significantly lower than visible ones (Fang and He, 2005; Hesselmann et al., 2011; Hesselmann and Malach, 2011; Ludwig et al., 2015).

For the dorsal stream however, the evidence is still not conclusive. One of the four abovementioned studies presented visible and invisible trials in separate runs without trial-by-trial subjective reports, and used long baseline conditions (Fang and He, 2005). They found that activity in dorsal areas was diminished for invisible faces but not for invisible tools. This dissociation may due to these two categories’ different relations to the function of reaching and grasping in the dorsal stream. By comparison, the other three studies presented visible and invisible trials in the same run, with trial-by-trial subjective reports. They all found higher activity for visible tools in both ventral and dorsal areas that covaried with the visual awareness (Hesselmann et al., 2011; Hesselmann and Malach, 2011; Ludwig et al., 2015). In two of them that performed multivariate pattern analyses between faces and tools, Hesselmann et al. (2011) found that invisible faces and tools were only decodable in the fusiform area, although Ludwig et al. (2015) found them decodable both in the right V3a/V7 in the dorsal stream, and in FFA in the ventral stream. A fifth study did not examine the amplitude of activity across the two streams, but specifically examined the decodability of faces and tools, with five different strengths of CFS masks. They found that the faces and tools were decodable in both streams with the no-mask condition and the weaker masks, associated with higher levels of subjective visibility but were not decodable for the stronger masks with lower levels of subjective visibility (Ludwig et al., 2016).

Here, we examined whether the assumed dissociation between the ventral and dorsal streams holds, and whether the ventral stream is mainly related to conscious and the dorsal stream to nonconscious perception, using stimuli of whole-body images. Human body stimuli have a unique combination of properties and are particularly useful to explore this issue, because they activate the action-related dorsal network (Rizzolatti and Sinigaglia, 2010) similarly to tool stimuli, and at the same time, bodies are processed in category specific areas (extrastriate body area, EBA; fusiform body area, FBA) in the ventral stream (Peelen and Downing, 2007). It has been shown that information of the body stimuli could be processed without visual awareness, through pathways other than V1, activating the EBA of the same patient with bilateral V1 lesions who showed object avoidance (Van den Stock et al., 2014). As a biologically meaningful category, the processing of bodies is also disrupted by the inversion of stimuli (Reed et al., 2003) similar to faces, and under the breaking CFS paradigm (b-CFS) which measures suppression time of stimuli and indirectly reflects the nonconscious processing, inverted bodies have been shown to be suppressed longer than upright bodies (Stein et al., 2012).

**Figure 1.**
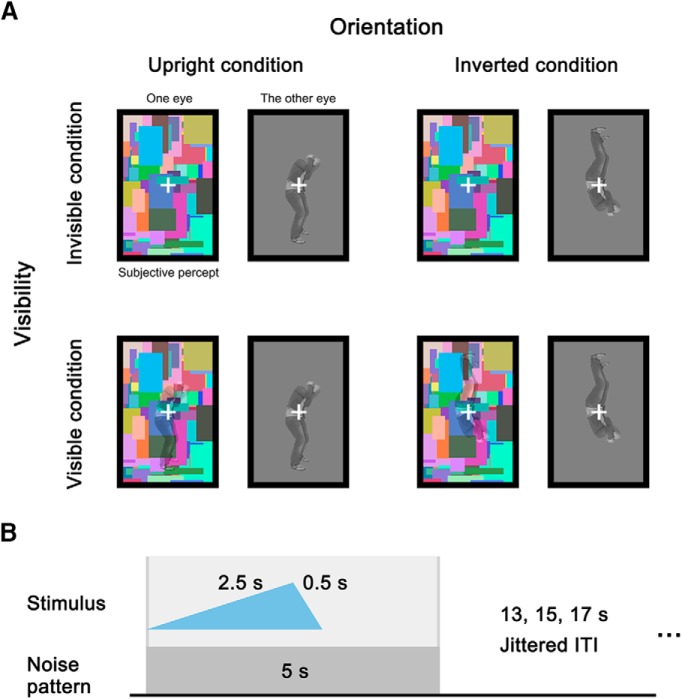
Stimulus presentation conditions and trial structure. ***A***, Four stimulus conditions of the factors visibility (visible, invisible) and orientation (upright, inverted). In each condition, a dynamic color noise pattern was presented in one eye, and the target stimulus was presented in the other eye. Participants could only subjectively perceive the contents in the rectangle with the dynamic noise. For visible conditions, the noise pattern was overlaid with the body stimulus. The dynamic noise was present in all conditions. ***B***, Structure of a single trial. The target stimulus was faded in for 2.5 s, and then faded out for 0.5 s. The contrast of the noise pattern was constant, and the noise pattern was present for another 2 s after the stimulus disappeared, to avoid perceiving afterimages. A jittered ITI followed the noise presentation. Gray vertical lines indicate the onset and offset of the stimulus presentation in a trial.

We presented the body stimuli either upright or inverted, and rendered them either invisible or visible with CFS, using a slow event-related design. By including inverted versions of exactly the same upright stimuli, the experimental design enabled us to examine the interaction of body orientation and subjective visibility, and to clarify the relationship between dorsal and ventral areas with respect to visual awareness. We measured blood-oxygenation level-dependent (BOLD) activity with fMRI of relatively high resolution (2 × 2 × 2 mm^3^), while participants passively viewed dichoptic stimuli through a pair of prism glasses. The four types of trials [orientation (upright, inverted) × visibility (visible, invisible)] were balanced and presented within the same runs (Fig. 1). We examined the relationships between orientation and visibility both with the general linear model (GLM) analysis and ANOVA in the whole brain, and performed the ANOVAs in ventral and dorsal regions of interest (ROIs) defined with a separate functional localizer for individual participants.

## Materials and Methods

### Participants

Eight participants took part in the current study, the data of seven were used for the analysis (two males, mean age = 25.7, SD = 4.1, 2 left-handed), the data of the other participant (participant 8) were excluded due to imperfect suppression (see the Validation of the suppression part in this section). The current experiment involved long scan sessions (2 h), thus continuing perfect CFS suppression is crucial; we applied stringent recruiting criteria on suppression effects for participants. Participants 2, 4, and 5 were recruited based on their performance in a separate CFS priming experiment using faces and bodies as prime stimuli where they did not perceive either faces or bodies (a total of 25 participants took part in this experiment at the time of invitation for the current fMRI study, for 16 of which the percept of the body stimuli was well suppressed, and for nine within the 16 the percept of the face stimuli was also suppressed). Participants 1, 3, 6, and 7 were colleagues in the department that had participated in various similar CFS pilot experiments with either suppressed faces or bodies. Participant 8 was recruited from another CFS experiment (20 participants in that experiment in total), where the visibility of body and object stimuli was manipulated in the same way as in the current fMRI experiment. For participant 8 the body stimuli were suppressed in 90.5% unseen trials in that experiment. This screening procedure also precluded any noticeable training effect of the task during the current fMRI experiment. As it was difficult to find participants with strong and stable suppression, we also included left-handed participants. Although all participants were familiar with the CFS paradigm, they were all naïve to the aim of the current study. The participants had no history of neurological disorders, had normal or corrected-to-normal visual acuity, and had normal stereoscopic color vision. They provided written informed consents for participation and received monetary rewards. The experimental procedures were approved by the ethical committee of Maastricht University, and were conducted in accordance with the standards established by the Declaration of Helsinki.

### The main experiment

#### Stimuli

Images of upright body postures expressing fear (24 identities, 12 were females) were selected from a validated set of whole-body stimuli (Stienen and de Gelder, 2011). To ensure successful suppression of stimuli during the scanning sessions, we used fearful body postures, because it was found in a previous behavioral study that fearful bodies were suppressed longer under CFS than neutral and angry bodies (Zhan et al., 2015). The bodies were aligned at the feet level, with facial information removed, and imbedded in a gray background (RGB value = 128,128,128, size = 240 × 160 pixels, 3.81 × 2.54° of visual angles). The bodies in the images had a height within 161 pixels (2.56°), and a width within 80 pixels (1.27°). The inverted body stimuli were created by turning the upright stimuli upside-down. For catch trials, the stimuli image contained one to four dots, randomly located in the image.

Dynamic Mondrian noise images with same size as the body stimuli were presented at 10 Hz, to achieve the suppression effect. The noise images contained colorful small rectangles (with height and width within 2°) that overlapped with each other; 600 unique noise images were created, and the images presented in each trial were randomly selected from this pool.

### Setup for dichoptic presentation

The dichoptic presentation both inside and outside the scanner was achieved by viewing the stimuli through a pair of prism glasses. Stimuli presentation was realized in MATLAB (the MathWorks) with Psychtoolbox (Brainard, 1997; Pelli, 1997). The dichoptic stimuli were presented into two rectangles (240 × 160 pixels) side by side, their centers displaced at equal distance from the center of the screen (792 pixels between centers of two rectangles, 12.41°). A frame of 10 pixels delineated the border of the rectangles, and a black fixation cross was placed in the center of each rectangle. A cardboard divider was positioned between the participant and the screen, dividing the distance between the two rectangles equally, to make sure that each eye of the participant only saw the rectangle ipsilateral to that eye. The diopters of the prism glasses were chosen according to the visual angles between the rectangles (Schurger, 2009). When viewing under this setup, the displacement for each rectangle would be removed by the prism glasses, thus shifting both of the rectangles back to the center of the screen. Participants were asked to free-fuse the two rectangles into one, using the frame and fixation cross of each rectangle. On successful fusion, participants would perceive a tunnel-like view, with the divider showing up on either side as the wall of the tunnel, and with one rectangle at the end of the tunnel in the center of the screen. Since the width of the perceived tunnel depended on the distance between the rectangles, to ensure the horizontal field of view of the gray background in the scanner was not too narrow for the participants, and for practical reasons (we have only one pair of prism glasses for each diopter), we used prism glasses of a bigger diopter (diopter = 12 for each eye) in the scanner, and a smaller diopter (diopter = 8 for each eye) outside the scanner. Apart from the distance between the two rectangles, other parameters of the experiment were kept the same both inside and outside the scanner. Participants reported no difficulty in merging the two rectangles into one, either inside or outside the scanner.

### Procedure of the main experiment

The main experiment used a slow event-related design. In each trial, a stimulus image was projected into one of the rectangular frames, and the dynamic noise was simultaneously projected into the other frame. The stimulus image was faded in from 0% to 50% contrast in 2.5 s, and subsequently faded out to 0% contrast in 0.5 s. The dynamic noise was presented at full contrast. To eliminate any possible afterimages of the stimulus, the dynamic noise was kept on the screen for another 2 s after the stimulus faded out. Each trial was followed by an interval of 13, 15, or 17 s. The fixation cross changed to white at 1 s before the start of each trial, remained white through the trial, and changed to black at the intertrial interval (ITI).

The experiment used a 2 × 2 factorial design, where stimuli were presented upright or inverted (orientation), invisible or visible (visibility). To keep the visible and invisible conditions as close as possible, the visible trials were created by overlaying the stimuli onto the noise pattern, resulting for all participants in a subjective percept of the body stimuli fading in and out with the presence of the noise pattern, which was a distinct percept from the invisible trials (percept of noise pattern only). To exclude confounds due to introspection and to motor response, we refrained from including a trial-by-trial report of the subjective percept.

For catch trials, the dot images were presented in both rectangles, similar to the body stimuli in the visible condition, so that the participants could see the dots fading in and out in the noise pattern. The trials were followed by a response screen for 2 s, indicated by a white circle replacing the fixation cross.

Participants were instructed to respond only to the dot trials, where they should indicate during the response screen presentation whether the number of the dots was odd or even, by pressing one of two corresponding buttons on a MR-compatible button box. For all the other trials, they were asked to fixate on the cross and passively view the presentation. The passive viewing task was used to avoid any confound of response-related activation, as could be observed in the parietal and frontal areas. Participants were also advised not to blink during the trials if possible, and to blink between the trials if needed.

The main scanning session consisted of four functional runs of 19 min and 10 s each. Within each run were 48 target trials (12 per condition) and 8 catch trials, presented in pseudorandom order. The side of the eyes that the dynamic noise projected into was also randomized and counterbalanced within the session. In total each individual stimulus was projected onto each eye twice: once visible and once invisible. For one participant, three functional runs were acquired; for the other participants, four functional runs were acquired. The anatomical scan was performed after two functional runs.

### Scanning parameters

The scanning was conducted in a Siemens 3T Prisma whole-body scanner (Siemens), with a 64-element head-neck coil. In the scanner, stimuli were back-projected with a LCD projector (Panasonic PT-EZ570, screen resolution = 1920 × 1200, refresh rate = 60 Hz) on a screen 75 cm away from the head of the participant. The cardboard divider was placed in the bore between the head coil and the screen. A T2*-weighted gradient echo EPI sequence was used to acquire functional data covering the whole brain, with 2 × 2 × 2 mm^3^ resolution (64 slices without gaps, TR (repetition time) = 2000 ms, TE (echo time) = 30 ms, flip angle = 77, simultaneous multi-slice acquisition acceleration factor = 2, FOV = 200 × 200, matrix size = 100 × 100). A T1-weighted MPRAGE sequence was used to acquire the anatomical structure images (1 × 1 × 1 mm^3^, TR = 2300 ms, TE = 2.98 ms).

### Validation of the suppression effect

For each participant, the effectiveness of suppression was validated by verbal reports during the scan after each run, and by behavioral validation runs before and after the scan. We based our decision of data selection mainly on the results of the behavioral validation runs.

To obtain online estimates of the CFS suppression efficiency during scanning, participants responded to the following three questions after each run. (1) In what percentage of trials did you see something in the noise? (2) Were there any merging problems during the scan? (3) Did you see a sudden appearance of the stimulus in the noise, rather than a gradual fading-in? A run with response of >60% seen trials (the actual percentage would be 57% when taking the seen catch trials into account), or sudden perception of stimulus in the noise, or any merging problem, would indicate that during the scan the suppression was not working perfectly. None of the runs included for data analysis had these problems.

The behavioral validation runs were conducted immediately before and after the scan, outside the scanner. The stimuli were presented on an LCD screen (Acer VG248, 3D capable, resolution = 1920 × 1080, refresh rate = 60 Hz), in a room with dim light. The distance between the two rectangles was adjusted according to the diopter of the prism glasses (276 pixels between centers of two rectangles, 9.15°, diopter = 8) to render stable fusion. Trials and their order in the runs before and after the scan corresponded to the run 1 and 2 in the scanner. There was no catch trial in the validation runs; instead a response screen with a circle (same as the one in the main experiment) was presented after stimulus presentation for each trial. Participants were required to respond whether they saw anything in the noise, by pressing either 1 (seen) or 2 (unseen) on the keyboard during the response screen on a trial-by-trial basis. If a participant responded “seen” for more than two times for the unseen (suppressed) trials in either one of the validation runs, including trials without response, the dataset of the participant would be excluded from analyses. The data of seven participants in this study satisfied the inclusion criterion (average accuracy for visible trials: 99.4%, average accuracy for invisible trials: 96.7%), showing that their subjective percept tightly followed our planned visibility manipulation. To further ensure that the stimuli were suppressed in the invisible trials during the fMRI scan, participants were asked again after the scan whether their visual experience of the stimuli was similar to that in the behavioral tasks before the scan. The percept of a stimulus escaping suppression (a stimulus suddenly appearing in the noise, instead of fading in slowly) was also clearly explained to the participants. All seven participants reported not having such percept. The 8th participant reported >70% seen trials after three fMRI runs in the scanner (with catch trials, reported 65–70%, 70–75%, and 60–70%, respectively), and responded three times “seen” for unseen trials in the behavioral test after scan (with no catch trials, reported percentage of seen trials: 50–60%, actual percentage 56%). Consistent with the behavioral test, after the scan this participant reported that in the behavioral test before she saw 50% trials (actual percentage = 52%, 1 trial breaking suppression), while reported “seeing more” in both the 2nd to 4th runs in the scanner and the behavioral test after scan. This participant was excluded from the analysis. The decrease of stimuli suppression efficiency for this participant might be the same effect reported by a few previous CFS studies, where participants saw more stimuli as the experiment progressed (Ludwig et al., 2013; Lupyan and Ward, 2013; Mastropasqua et al., 2015; Stein and Peelen, 2015).

In total, 26 runs (575 volumes each) from seven participants were included in the analysis. One run from another participant was excluded, due to merging problems caused by a contact lenses issue that occurred during that run. Another participant completed three runs instead of four runs.

### Functional localizer

Participants were also scanned with a functional localizer run (432 volumes) in a separate session, where they passively viewed stimuli of faces, bodies, houses, tools and words in blocks. Facial stimuli were front-view neutral faces from the Karolinska Directed Emotional Faces (Lundqvist et al., 1998; 24 identities, 12 males). The part below the neck (clothes, hair, etc.) was removed from the face images. Body stimuli (de Gelder and Van den Stock, 2011) were neutral still front-view bodies different from the ones used in the main experiment (20 identities, 10 males), with the facial information removed. House and tool images were obtained from the Internet. The house images consisted of 19 facades of houses with two-to-three-storey height, and the tool images consisted of 18 hand-held tools. Words images consisted of high-frequency English words of four to six letters in Arial font. All the images were imbedded within a gray background (RGB value = 157,157,157), spanning a visual angle of 3.65 degrees (230 pixels). Each block consisted of 12 stimuli from the same category; each stimulus was presented for 800 ms, followed by an interval of 200 ms. An interblock interval of 12 s followed each block presentation. Blocks of each category were presented seven times, and the presentation order of the stimuli and the blocks were pseudorandomized.

### Data processing

The acquired data were processed in BrainVoyager (Brain Innovation). Functional data underwent default slice scan time correction, 3D motion correction, temporal GLM with Fourier basis high-pass filtering of two cycles. The functional datasets were then aligned to the anatomical images, brought into Talairach space, and underwent spatial smoothing with a Gaussian filter of 4-mm FWHM.

### GLM analyses

Random effects group analyses with GLM were applied to the functional data of the main experiment. Predictors for each condition were convolved with the default two-γ hemodynamic response function. The parameters from 3D head motion correction were z-transformed and added as confound predictors into the GLM analyses. The percentage signal change values for each participant were extracted for subsequent ROI analyses. A 2 × 2 ANOVA with orientation and visibility was performed on the whole-brain basis. To observe the configural processing of fearful bodies, contrast of upright invisible > inverted invisible was also performed. The clusters of the ANOVA and the contrast analyses were corrected for multiple comparison by cluster threshold estimation (initial threshold *p* = 0.005 for the ANOVA results, initial threshold *p* = 0.01 for the contrast results, Monte Carlo simulation *n* = 5000).

### ROI analyses

**Figure 2.**
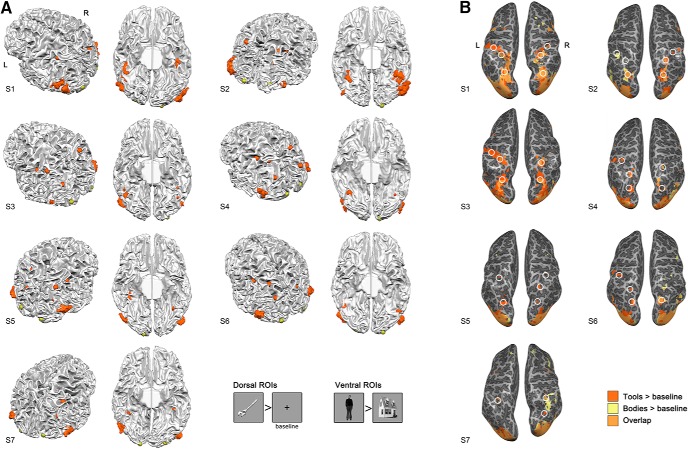
Definition of ROIs in individual participants, shown in neurological view. ***A***, Ventral, dorsal and V1 ROIs in individual participants, defined by separate functional localizer data. Ventral ROIs (orange color, irregular shape) were defined by the contrast bodies > houses (*p* = 0.001 uncorrected); dorsal spherical ROIs (orange color, spherical shape) were drawn at the peak activation sites of the contrast tools > baseline (the fixation cross, *p* = 0.001 uncorrected), in the posterior, middle, and anterior branches of IPS, respectively. V1 spherical ROIs (yellow color) were defined inside bilateral occipitopolar sulci. Participants S2 and S5 are left-handed. The dorsal views are shown in the angle and side where most of the dorsal ROIs could be seen. Some of the dorsal ROIs could not be defined in all participants. The ventral view shows the ventral ROIs. If the clusters in either EBA or FBA consisted of multiple smaller clusters, they were grouped into one. The example stimuli of the functional localizer and the contrasts are shown in the lower right corner. ***B***, The areas activated by bodies and tools largely overlapped, especially in the posterior part of the IPS. Areas shown were: tools > baseline (*p* = 0.001 uncorrected), bodies > baseline (*p* = 0.001 uncorrected). The locations for dorsal ROIs were marked with white circles.

Functional ROIs were defined by GLM contrasts on the functional localizer data, individually for each participant (Fig. 2). Ventral ROIs were defined by the contrast bodies > houses (*p* = 0.001 uncorrected). Clusters that were located in the lateral occipital sulcus were marked as EBA; clusters located in the fusiform region were marked as FBA. Dorsal ROIs were defined for the anterior, middle and posterior intraparietal sulcus (IPS) bilaterally, by contrasting tools > baseline (*p* = 0.001 uncorrected). Spheres (radius = 4 mm) were defined at the peak activation sites located in the anterior (connecting postcentral sulcus), middle, and posterior segments of IPS, respectively. As a comparison to the ventral and dorsal areas, sphere ROIs of the primary visual cortex (V1) were defined at the occipital pole, at the spots in bilateral occipitopolar sulci where the calcarine sulci pointed to (radius = 4mm). The V1 ROIs defined anatomically were located within the extensive cluster activated by visual presentation of the 5 conditions in the functional localizer versus baseline (*p* = 0.00001, uncorrected). For FBA, EBA, pIPS, mIPS, aIPS, and V1, we performed a group-level ANOVA of ROI (six areas) × laterality (left, right) × orientation × visibility, where for each unilateral ROI one averaged percentage signal change value per participant was entered as input. This group-level ANOVA did not show either a significant main effect of laterality (*F*_(1,1)_ = 2.453, *p* = 0.362), or interactions with laterality (ROI × laterality: *F*_(5,5)_ = 0.550, *p* = 0.736; laterality × orientation: *F*_(1,1)_ = 3.903, *p* = 0.298; ROI × laterality × orientation: *F*_(5,5)_ = 1.048, *p* = 0.480; laterality × visibility: *F*_(1,1)_ = 0.606, *p* = 0.579; laterality × orientation × visibility: *F*_(1,1)_ = 0.537, *p* = 0.597; ROI × laterality × orientation × visibility: *F*_(5,5)_ = 1.430, *p* = 0.352). Thus, we merged the bilateral ROI pairs into single ROIs. For some of the dorsal areas, only unilateral ROIs could be defined in some participants (e.g., the right aIPS could only be defined in three participants), in those cases the data of the unilateral ROI were entered into further analysis. To compare the ventral and dorsal ROIs directly, the bilateral FBA and EBA ROIs were merged into one combined ventral ROI, and the bilateral ROIs along the IPS were merged into one combined dorsal ROI. The mean percentage signal change values from the GLM analysis were extracted for each resulting ROI of each participant. Group-level repeated-measures ANOVAs were performed in SPSS. We first conducted an ANOVA of stream (ventral, dorsal) × orientation (upright, inverted) × visibility (visible, invisible) with the data of the combined ventral and dorsal ROIs. In case that an interaction was present, we examined the orientation × visibility ANOVA in the specific stream, then conducted subsequent ANOVAs with the data of individual ROIs.

### ROI analysis in individual participants

To rule out that the observed results of group-level ANOVAs in our ROI analysis were driven by a minority of participants, we performed within-participant ROI analysis in the seven individual participants, examining the prevalence of effects (or no effects in the dorsal stream). To be most comparable to the group-level ROI analysis, we fitted the same GLM to each run in individual participants. The percentage signal changes (parameter estimates) of each condition were extracted from the same bilateral ROIs of the ROI analysis (including the combined ventral and dorsal ROIs, and the six individual ROIs), in individual participants, and entered into within-participant repeated-measures ANOVAs. Because the number of runs was different across participants, the number of parameter estimates included in the ANOVAs was different (three estimates per condition in participants S1 and S3, four estimates per condition in all five other participants). The ANOVAs included the stream × orientation × visibility ANOVA in the ventral/dorsal combined ROIs, and the orientation × visibility ANOVAs in the six individual ROIs. Lastly, to compare with the results obtained in Fang and He (2005), we performed the pairwise comparisons of upright visible versus upright invisible conditions in these eight ROIs.

## Results

### Whole-brain analysis

**Figure 3.**
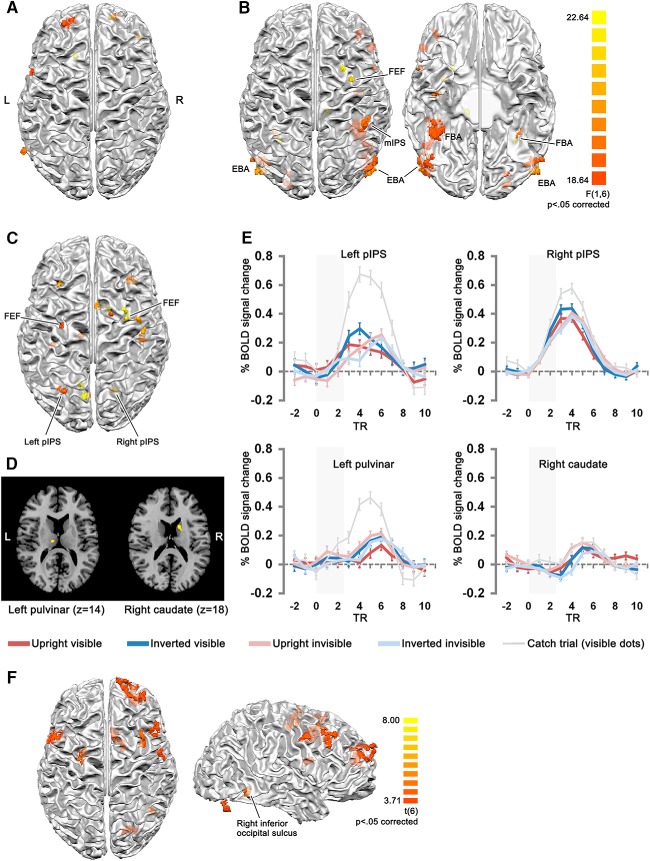
Clusters of the group-level whole-brain ANOVA, and contrast analysis of upright invisible > inverted invisible (cluster size corrected, initial threshold *p* = 0.005 for the ANOVA, *p* = 0.01 for the contrast, Monte Carlo simulation *n* = 5000), projected onto the 3D surface of white-gray matter boundary of one participant, shown in neurological view. ***A***, Clusters showing a main effect of orientation (upright, inverted). ***B***, Clusters showing a main effect of visibility (visible, invisible). ***C***, Clusters showing the interaction of orientation and visibility. ***D***, The subcortical clusters from ***C*** (in-slice neurological view). The color bar for the clusters in ***A****–****D*** is shown in ***B*. *E***, The percentage signal change of the clusters in ***C***, ***D***, for the left and right pIPS, the left pulvinar, and the right caudate clusters. Gray areas in the time course plots indicate the duration of dynamic noise presentation. The next trial started at TR 9-11 with a jittered ITI (13, 15, or 17 s after the offset of the dynamic noise). The time courses for catch trials (visible dots, requiring participants to respond by button pressing after dynamic noise presentation) were plotted with gray thin lines, as a comparison. Note that the time courses for catch trials have higher percentage-signal change than the main conditions. Error bars denote SEM. ***F***, Clusters shown by the contrast upright invisible > inverted invisible. The lateral view of the brain provides a clearer view of the two posterior clusters (one in the right inferior occipital sulcus and another in the right cerebellum, shown outside the cortex mesh).

We conducted a whole-brain ANOVA at the group level, with orientation (upright, inverted) and visibility (visible, invisible) as factors (Fig. 3; Table 1). The main effect of orientation (upright, inverted) was observed in clusters mainly in the frontal lobe, and a cluster close to the EBA region defined with the functional localizer. A main effect of visibility (visible, invisible) was observed mainly in clusters in the ventral pathway, including bilateral EBA, FBA, lateral occipitotemporal cortex, and right anterior inferior temporal cortex. Clusters in the dorsal pathway were located in bilateral anterior IPS, and right middle frontal gyrus (corresponding to the frontal eye field, FEF). Other clusters were located at the right inferior frontal lobe, and right posterior cingulate sulcus.

**Table 1.** List of clusters shown by the whole-brain ANOVA

Region	Tal *X*	*Y*	*Z*	SD *x*	SD *y*	SD *z*	Size (mm^3^)	Peak *x*	Peak *y*	Peak *z*	Peak *F*	Peak *p*
Visibility × orientation interactions												
R central sulcus	41.88	−20.88	54	1.54	1.69	1.12	78	43	−21	54	41.895	0.0006
R central sulcus	43.3	−13.75	52.39	1.02	1.4	1.06	56	43	−14	52	39.4	0.0008
R inferior precentral sulcus	39.34	−4.53	51.5	1.69	1.35	1.74	116	39	−4	52	44.075	0.0006
R middle frontal sulcus, anterior	32.79	28.8	34.09	2.22	1.53	2.12	178	31	29	35	88.547	8E-05
R superior frontal sulcus	29.47	−2.43	53.41	1.27	1.34	1.14	79	30	−2	54	65.446	0.0002
R superior frontal sulcus	27.61	3.46	62.25	1.32	1.32	1.24	59	27	4	63	39.093	0.0008
R posterior IPS (connecting to the mIPS)	18.2	−64.4	38.09	1.66	1.1	1.35	82	17	−64	39	49.916	0.0004
R superior frontal gyrus	15.95	0.19	64.86	0.96	0.79	2.15	64	16	0	66	43.332	0.0006
R caudate	13.82	4.62	17.39	1.42	2.14	0.84	74	13	6	17	53.046	0.0003
R posterior cingulate sulcus	4.15	7.9	45.11	1.25	1.64	1.86	88	3	7	47	43.572	0.0006
L precuneus, inferior	−3.76	−69.67	36.48	1.09	0.96	1.51	54	−3	−69	36	69.414	0.0002
L precuneus, superior	−7.26	−69.85	45.47	1.48	1.63	0.98	95	−8	−70	46	41.893	0.0006
L pulvinar	−9.71	−17.89	13.16	1.47	0.98	0.98	56	−10	−18	13	33.34	0.0012
L superior parietal gyrus	−10.41	−60.28	58.01	1.14	1.71	2.48	75	−10	−62	56	72.816	0.0001
L posterior IPS	−25.1	−64.65	56.47	2.19	1.43	1.31	104	−24	−65	57	49.793	0.0004
L middle frontal gyrus	−26.35	−9.48	58.7	1.02	1.27	1.26	54	−27	−10	59	33.024	0.0012
L middle frontal sulcus/superior frontal sulcus	−27.89	26.62	38.79	1.01	1.3	1.53	61	−28	27	38	43.902	0.0006
L posterior insula	−33.76	−25.47	22.4	1.04	0.97	1.26	58	−34	−26	23	56.325	0.0003
Main effect orientation												
R middle frontal gyrus	38.47	38.18	22.25	1.35	1.04	1.25	51	38	38	22	33.466	0.0012
R superior frontal gyrus, anterior	18.33	55.51	26.98	0.94	1.39	0.96	55	18	56	27	36.501	0.0009
R superior frontal sulcus, anterior	14.96	54.72	6.28	1.22	0.92	1.92	72	14	54	5	66.051	0.0002
L anterior cingulate sulcus	−15.02	21.98	31.78	1.16	1.1	1.12	59	−15	22	32	89.787	8E-05
L superior frontal sulcus/gyrus	−21.33	50.2	31.18	3.47	2.41	2.57	306	−25	49	32	59.614	0.0002
L superior frontal sulcus/gyrus	−21.66	52.66	14.17	0.88	1.61	1.03	59	−22	52	14	36.22	0.0009
L inferior frontal sulcus and inferior precentral sulcus, connection point	−52.39	8.62	34.87	1.04	1.63	1.48	69	−53	7	35	36.063	0.001
L lateral temporal gyrus (anterior to the EBA found in 6 participants, overlapping with 1)	−59.31	−58.91	0.67	1.23	1.55	1.19	91	−59	−60	1	49.214	0.0004
Main effect visibility												
R lateral occipital sulcus (EBA)	49.1	−70.36	16.38	2.73	2.85	3.49	400	49	−69	18	51.073	0.0004
R inferior frontal gyrus, pars opercularis	50.75	11.83	13.1	1	1.14	1.19	59	51	12	13	51.765	0.0004
R lateral occipital sulcus (EBA)	47.7	−77.04	12.61	1.94	1.7	1.8	148	46	−78	13	57.856	0.0003
R inferior frontal gyrus, pars obitalis	48.53	28.97	−2.96	1.7	1.56	1.67	154	47	28	−4	47.69	0.0005
R lateral occipital sulcus (EBA)	47.55	−61.26	12.18	1.29	0.88	1.74	76	48	−61	12	43.297	0.0006
R inferior occipital gyrus (Lateral occipital complex)	44.39	−71.09	−10.71	2.23	4.49	6.14	516	46	−70	−16	45.839	0.0005
R fusiform gyrus (FBA)	38.62	−44.05	−16.4	3.99	6.48	2.04	1050	38	−44	−18	123.67	3E-05
R IPS, anterior branch	39.93	−37.16	46.85	3.57	3.15	2.79	525	39	−41	49	92.748	7E-05
R inferior frontal gyrus, pars obitalis	37.37	40.1	10.15	1.46	2.13	1.44	157	37	39	9	61.047	0.0002
R collateral gyrus, anterior (anterior temporal lobe)	36.74	−10.13	−28.58	2.14	1.15	0.98	89	35	−9	−29	65.553	0.0002
R middle frontal gyrus (FEF)	30.73	1.75	63.45	1.09	1.18	0.94	56	31	2	64	70.458	0.0002
R superior frontal sulcus	23.78	10.87	57.48	1.26	1.28	0.81	54	24	11	57	45.353	0.0005
R posterior cingulate sulcus	10.69	−25.79	42.11	1.07	1.39	1.17	62	11	−26	42	43.09	0.0006
L fourth occipital gyrus	−23.27	−88.1	−8.6	2.29	1.28	0.93	97	−25	−87	−9	127.47	3E-05
L posterior collateral sulcus	−22.52	−78.63	−9.81	1.22	1.23	1.04	63	−22	−79	−10	31.852	0.0013
L IPS, connection point of the middle branch and the anterior branch	−30.19	−51.12	40.18	1.13	1.23	1.25	73	−30	−51	40	63.971	0.0002
L collateral sulcus (anterior fusiform region)	−34.12	−43.66	−16.2	1.71	2.17	0.71	59	−34	−44	−16	25.157	0.0024
L inferior occipital sulcus (EBA)	−45.19	−68.53	−6.68	4.17	2.18	1.62	337	−40	−71	−8	70.051	0.0002
L lateral occipital sulcus (EBA)	−46.95	−76.46	−0.25	2.47	1.52	1.51	222	−45	−77	−1	78.08	0.0001
Contrast: upright invisible >inverted invisible												
R precentral sulcus	50.41	−2.81	43.21	2.07	2.84	1.97	172	48	−2	44	7.1546	0.0004
R middle frontal gyrus	45.89	20.4	45.28	1.65	4.77	1.7	194	45	25	45	6.5412	0.0006
R middle frontal gyrus	42.49	19.05	37.05	2.6	1.73	3.25	238	41	18	34	6.1623	0.0008
R inferior occipital sulcus	42.31	−55.71	−7.34	1.6	1.57	1.98	151	42	−56	−7	7.2599	0.0003
R superior frontal sulcus/R superior frontal gyrus, anterior	24.58	52.78	24.29	6.43	6.45	4.8	1340	32	48	21	8.1961	0.0002
R middle frontal gyrus (FEF)	32.99	9.7	48.79	1.64	4.32	1.69	243	34	5	48	5.8615	0.0011
R cerebellum	20.5	−72.99	−24.53	3.77	1.76	2.78	240	19	−72	−27	7.4966	0.0003
R caudate	14.79	3.99	17.44	1.34	2.68	1.43	151	15	1	16	9.4184	8E-05
R medial frontal gyrus	6.86	15.9	56.65	1.91	3.48	1.85	161	5	19	55	5.0247	0.0024
L superior frontal sulcus (FEF)	−22.89	−5.04	53.37	1.61	3.29	5.05	377	−24	−4	55	6.5383	0.0006
L inferior frontal sulcus and inferior precentral sulcus,connection point	−48.15	13.04	36.09	3.88	2.83	3.84	424	−48	13	37	7.7126	0.0002

Importantly, the interaction of visibility and orientation was observed mainly in clusters of the parietal and frontal cortex, that overlap with regions of the dorsal attention network (Corbetta and Shulman, 2011). The parietal clusters included left medial IPS, left precuneus, right posterior IPS. The frontal clusters were located along bilateral superior frontal sulci, mostly at the location of FEF, but also more anteriorly for two clusters. Another cluster was located in the right anterior cingulate sulcus, close to the presupplementary motor area. Importantly, the interaction effect also revealed clusters in subcortical areas, including the left pulvinar and the right caudate nucleus. When mirrored to the right hemisphere, the coordinates of these two clusters corresponded to the focal lesion sites found in spatial neglect patients with restricted subcortical lesions (Karnath et al., 2002).

We also conducted a whole-brain contrast of upright invisible > inverted invisible, which showed clusters mainly in the frontal lobe. Importantly, a cluster was present in the right inferior occipital sulcus, showing higher activity for upright bodies. This indicates that despite being invisible, the upright bodies were nonetheless processed more extensively than the inverted ones in the ventral pathway. A cluster was also present in the right caudate nucleus.

### ROI analysis

The functional localizer included still images of faces, bodies, houses, tools and words. We defined ventral ROIs by the bodies > houses contrast (*p* = 0.001 uncorrected), leading to ROIs of bilateral EBA and FBA. Because the tools activate dorsal action observation and execution related structures, we defined the dorsal ROIs by the tools > baseline contrast (*p* = 0.001 uncorrected). The areas activated by tools largely overlapped with those activated by bodies (bodies > baseline, *p* = 0.001 uncorrected), especially at the posterior IPS. For the overlaps in individual participants, see Figure 2. Sphere ROIs of 4mm radius were defined at the peak activation sites in the anterior (connecting postcentral sulcus), middle, and posterior segments of IPS, respectively (labeled aIPS, mIPS, and pIPS). For comparison with the ROIs in the ventral and dorsal streams, we also defined sphere ROIs in the bilateral primary visual cortex (V1) that was activated by visual presentation of these 5 stimuli categories. For ROIs of individual participants, see Figure 2. Because we did not find main effects or interactions related to the laterality factor in the group-level ANOVA of areas (six ROI pairs) × laterality (left, right) × orientation × visibility, we merged the bilateral ROIs in each area into one ROI, and then combined the ventral and dorsal ROIs, respectively, to directly examine whether the dorsal stream areas indeed show a different response pattern than the ventral stream.

First, we performed a repeated-measures ANOVA of stream (ventral/dorsal) × orientation × visibility on the averaged percentage signal changes of the combined ventral ROI and the combined dorsal ROI. If the response patterns differ between the two streams across the conditions, it would lead to an interaction of stream × visibility. Indeed, we found a significant interaction of stream × visibility (*F*_(1,6)_ = 30.821, *p* = 0.001, η_p_^2^ = 0.837), and a significant interaction of stream × orientation × visibility (*F*_(1,6)_ = 7.307, *p* = 0.035, η_p_^2^ = 0.549), in line with our prediction. The main effect of visibility was also significant (*F*_(1,6)_ = 33.370, *p* = 0.001, η_p_^2^ = 0.848). We subsequently performed the orientation × visibility ANOVA with the averaged activity separately for each stream.

**Figure 4.**
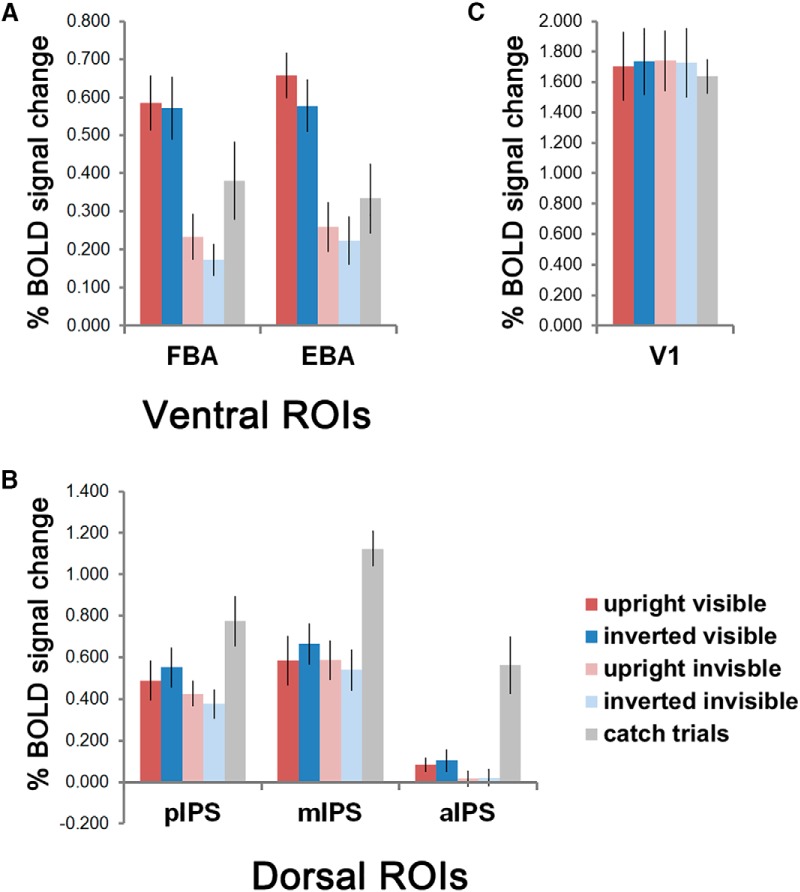
The average percentage signal change for the four main conditions and the catch trials (judging number of visible dots, followed by a button press) in ventral, dorsal, and V1 ROIs. ***A***, Ventral ROIs (*n* = 7). ***B***, Dorsal ROIs (*n* = 7 for pIPS and mIPS, *n* = 6 for aIPS). ***C***, V1 ROIs (*n* = 7). Error bars denote SEM. See Extended Data Figure 4-1 for percentage signal changes data of individual participants.

The combined ventral ROI showed strong main effects of orientation, and visibility, with no interaction effect. Similar to the ventral clusters shown by the main effect of visibility in the whole-brain ANOVA, visible bodies consistently elicited higher activity than suppressed invisible bodies (*F*_(1,6)_ = 38.063, *p* = 0.001, η_p_^2^ = 0.864), which is in accordance with the findings in CFS studies using other stimulus categories (Fang and He, 2005; Jiang and He, 2006; Hesselmann and Malach, 2011; Yang et al., 2014). Upright bodies also elicited higher activity than inverted ones (*F*_(1,6)_ = 16.297, *p* = 0.007, η_p_^2^ = 0.731), also consistent with studies using other categories of inverted stimuli, such as faces (Pinsk et al., 2009; Gilaie-Dotan et al., 2010). See Table 2 for the statistical results of the ANOVA. We also examined the averaged percentage signal changes for each condition in the FBA and EBA ROIs separately (Fig. 4; Table 2). Notably, the reduced activation for inverted bodies was consistent across visibility conditions, as a main effect of orientation was found in both the FBA and EBA ROIs (FBA: *F*_(1,6)_ = 9.950, *p* = 0.020, η_p_^2^ = 0.624; EBA: *F*_(1,6)_ = 13.230, *p* = 0.011, η_p_^2^ = 0.688), without interaction effects to visibility. For the invisible conditions, *post hoc* paired *t test* showed significantly higher activity for the upright bodies in the FBA ROI (*t*_(6)_ = 3.111, *p* = 0.021), and a trend to significance in the EBA ROI (*t*_(6)_ = 2.154, *p* = 0.075). Together with the activation in right inferior occipital gyrus observed under the contrast upright invisible > inverted invisible in the whole-brain analysis, this ROI result shows that ventral body-specific areas are sensitive to the orientation of body stimuli even when the bodies are presented without visual awareness.

10.1523/ENEURO.0285-17.2017.f4-1 Extended Data Figure 4-1 The average percent signal change data for the four main conditions and the catch trials (judging number of visible dots, followed by a button press) for individual participants. Download Figure 4-1, XLSX file.

**Table 2.** Results of ANOVAs in combined ventral and dorsal ROIs, and in ROIs of individual areas

ROIs	ANOVA		*F*	*p*	η_p_^2^
Combined ventral ROI, combined dorsal ROI	Stream × orientation × visibility	Stream	0.013	0.913	0.002
	*n* = 7	Orientation	2.006	0.206	0.251
		Visibility	33.370	*0.001	0.848
		Stream × orientation	5.296	0.061	0.469
		Stream × visibility	30.821	*0.001	0.837
		Orientation × visibility	2.231	0.186	0.271
		Stream × orientation × visibility	7.307	0.035	0.549
	Stream × visibility (upright conditions only)	Stream	0.098	0.765	0.016
	*n* = 7	Visibility	24.987	*0.002	0.806
		Stream × visibility	34.612	*0.001	0.852
Combined ventral ROI	Orientation × visibility	Orientation	16.297	*0.007	0.731
	*n* = 7	Visibility	38.063	*0.001	0.864
		Orientation × visibility	0.425	0.538	0.066
Combined dorsal ROI	Orientation × visibility	Orientation	0.122	0.738	0.02
	*n* = 7	Visibility	9.172	*0.023	0.605
		Orientation × visibility	13.624	*0.01	0.694
Dorsal ROIs: pIPS, mIPS, aIPS	Area × orientation × visibility	Area	9.962	*0.004	0.666
	*n* = 6	Orientation	0.192	0.679	0.037
		Visibility	6.149	0.056	0.552
		Area × orientation	0.131	0.879	0.026
		Area × visibility	0.847	0.457	0.145
		Orientation × visibility	10.853	*0.022	0.685
		Area × orientation × visibility	9.449	*0.005	0.654
ROIs of individual areas	ANOVA		*F*	*p*	η_p_^2^
V1	Orientation × visibility	Orientation	0.628	0.458	0.095
	*n* = 7	Visibility	0.174	0.691	0.028
		Orientation × visibility	2.882	0.14	0.324
FBA	Orientation × visibility	Orientation	9.950	*0.02	0.624
	*n* = 7	Visibility	37.446	*0.001	0.862
		Orientation × visibility	1.780	0.231	0.229
EBA	Orientation × visibility	Orientation	13.230	*0.011	0.688
	*n* = 7	Visibility	35.008	*0.001	0.854
		Orientation × visibility	1.223	0.311	0.169
pIPS	Orientation × visibility	Orientation	0.056	0.821	0.009
	*n* = 7	Visibility	7.477	*0.034	0.555
		Orientation × visibility	19.060	*0.005	0.761
mIPS	Orientation × visibility	Orientation	0.201	0.669	0.032
	*n* = 7	Visibility	2.711	0.151	0.311
		Orientation × visibility	10.111	*0.019	0.628
aIPS	Orientation × visibility	Orientation	0.202	0.672	0.039
	*n* = 6	Visibility	10.812	*0.022	0.684
		Orientation × visibility	0.689	0.444	0.121

Significant results are indicated with *.

In the combined dorsal ROI, the ANOVA of orientation × visibility again showed a main effect of visibility (*F*_(1,6)_ = 9.172, *p* = 0.023, η_p_^2^ = 0.605). Important, however, it also showed an interaction of orientation × visibility (*F*_(1,6)_ = 13.624, *p* = 0.010, η_p_^2^ = 0.694). To directly compare our results to other CFS studies without manipulation of stimulus orientation, we also performed the ANOVA stream × visibility with only the upright conditions. Again a strong interaction of stream × visibility was observed (*F*_(1,6)_ = 34.612, *p* = 0.001, η_p_^2^ = 0.852), together with the main effect of visibility (*F*_(1,6)_ = 24.987, *p* = 0.002, η_p_^2^ = 0.806). To better understand the interaction effects found in the dorsal stream, we performed an ANOVA of area (pIPS, mIPS, and aIPS) × orientation × visibility. Again we found the interaction orientation × visibility (*F*_(1,5)_ = 10.853, *p* = 0.022, η_p_^2^ = 0.685), but we also found a significant main effect of area (*F*_(2,10)_ = 9.962, *p* = 0.004, η_p_^2^ = 0.666), and a strong interaction of area × orientation × visibility (*F*_(2,10)_ = 9.449, *p* = 0.005, η_p_^2^ = 0.654), indicating that the response patterns changed across the areas within the dorsal stream. The main effect of visibility showed a trend toward significance (*F*_(1,5)_ = 6.149, *p* = 0.056, η_p_^2^ = 0.552). Indeed, separate inspections of the activity in pIPS, mIPS, and aIPS ROIs showed that the interaction effect of orientation × visibility was present in both pIPS and mIPS ROIs, but was not present in the aIPS ROI, which showed a main effect of visibility instead, with higher activity for upright than inverted bodies, similar to the pattern of the ventral areas. For the pIPS ROI, the main effect of visibility was also present. In both the pIPS and mIPS ROIs, *post hoc* paired *t* tests showed that the activity between visible and invisible upright bodies did not differ (pIPS: *t*_(6)_ = 1.166, *p* = 0.288; mIPS: *t*_(6)_ = −0.040, *p* = 0.970), but the activity between the two inverted conditions differed (pIPS: *t*_(6)_ = 4.886, *p* = 0.003, mIPS: *t*_(6)_ = 4.630, *p* = 0.004; Fig. 4; Table 2).

In comparison to the ventral and dorsal ROIs, no significant main effect or interaction was observed for V1 ROIs (all p>.05).

### ROI analysis in individual participants

**Figure 5.**
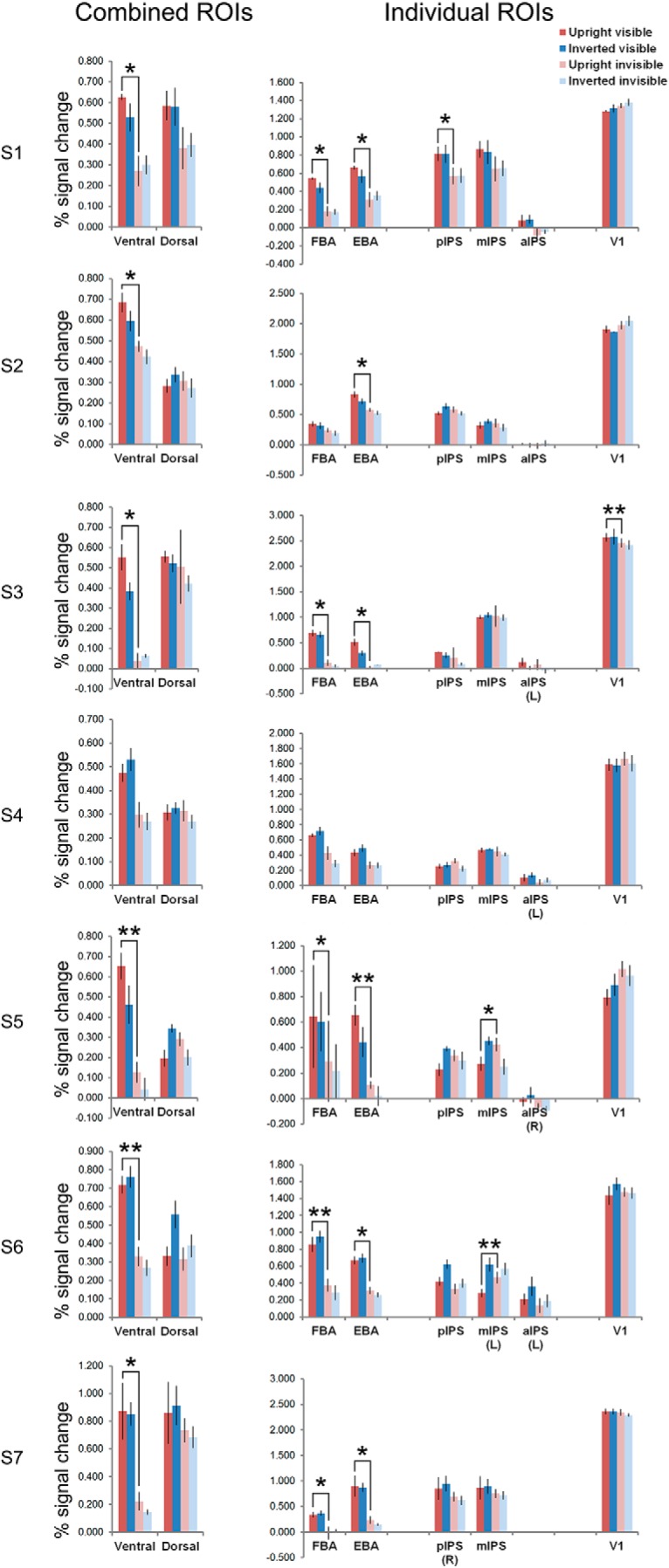
The average percentage signal change for the four main conditions in the combined ventral and dorsal ROIs, and individual ROIs, at single-participant level. Unless marked, all ROIs included voxels from bilateral areas. Repeated-measures ANOVAs were performed in individual participants, with average percentage signal changes (parameter estimates of the GLM) per run per condition as inputs. Error bars denote SEM. For S1 and S3 the number of parameter estimates was three per condition, for the other five participants the number of parameter estimates was four per condition. Participants S2 and S5 are left-handed. The significant pairwise comparisons between upright visible and upright invisible conditions were plotted; *p < 0.05, **p < 0.01. Note that most of the dorsal ROIs did not show significant activity differences between upright visible and upright invisible conditions. Furthermore, the significant effects in dorsal ROIs had opposite directions to the ones found in ventral ROIs. See Table 3 for the *p* values of the ANOVAs and pairwise comparisons.

To rule out the possibility that the abovementioned ROI results were driven by a minority of participants, we performed within-participant repeated-measures ANOVAs in the bilateral ROIs of the seven individual participants. See Figure 5 and Table 3 for averaged responses per condition, the *p* values for the statistical tests, and the directions of significant main effects.

**Table 3.** *p* Values of the ANOVAs and pairwise comparisons for within-participant analysis in ROIs of individual participants

Participant		S1	S2	S3	S4	S5	S6	S7	Direction of effect (number of participants showing an effect)
Number of runs		3	4	3	4	4	4	4	
Ventral/dorsal ROIs × orientation × visibility	Stream	0.190	0.002*	0.066*	0.027*	0.346	0.024*	0.011*	Ventral > dorsal (3)Dorsal > ventral (1)
	Orientation	0.742	0.515	0.257	0.990	0.429	0.084	0.746	
	Visibility	0.049*	0.089	0.100	0.011*	0.005*	0.00003*	0.045*	Visible> invisible (5)
	Stream × orientation	0.541	0.010*	0.878	0.581	0.010*	0.010*	0.307	(3)
	Stream × visibility	0.022*	0.002*	0.018*	0.002*	0.002*	0.0003*	0.001*	(7)
	Stream × orientation × visibility	0.337	0.050	0.300	0.632	0.008*	0.374	0.591	(1)(trend 1)
Individual ROIs									
FBA	Orientation	0.211	0.459	0.141	0.304	0.730	0.970	0.757	
	Visibility	0.024*	0.146	0.018*	0.0004*	0.010*	0.00005*	0.015*	Visible > invisible (6)
	Orientation × visibility	0.365	0.843	0.854	0.217	0.719	0.261	0.886	
EBA	Orientation	0.114	0.141	0.060	0.383	0.107	0.755	0.542	
	Visibility	0.053	0.012*	0.034*	0.005*	0.004*	0.00008*	0.009*	Visible > invisible (6) (trend 1)
	Orientation × visibility	0.323	0.544	0.035*	0.527	0.162	0.541	0.729	(1)
pIPS	Orientation	0.974	0.714	0.491	0.260	0.466	0.004*	0.760	Inverted > upright (1)
	Visibility	0.047*	0.618	0.390	0.525	0.857	0.005*	0.193	visible > invisible (2)
	Orientation × visibility	0.975	0.032*	0.783	0.099	0.129	0.169	0.267	(1)
mIPS	Orientation	0.898	0.952	0.964	0.803	0.950	0.003*	0.972	Inverted > upright (1)
	Visibility	0.147	0.444	0.870	0.203	0.692	0.027*	0.425	Invisible > visible (1)
	Orientation × visibility	0.894	0.089	0.849	0.654	0.011*	0.025*	0.685	(2)
aIPS	Orientation	0.551	0.532	0.028*	0.385	0.804	0.131		Upright > inverted (1)
	Visibility	0.029*	0.968	0.391	0.201	0.169	0.038*		Visible > invisible (2)
	Orientation × visibility	0.916	0.453	0.987	0.970	0.468	0.583		
V1	Orientation	0.315	0.350	0.887	0.740	0.712	0.267	0.707	
	Visibility	0.075	0.106	0.101	0.252	0.014*	0.556	0.091	Invisible > visible (1)
	Orientation × visibility	0.866	0.351	0.674	0.130	0.374	0.285	0.702	
Pairwise comparison of upright visible and upright invisible	Ventral	0.031*	0.020*	0.033*	0.083	0.005*	0.008*	0.033*	Upright visible > upright invisible(6)
	Dorsal	0.131	0.360	0.830	0.894	0.068	0.739	0.581	
	FBA	0.012*	0.105	0.034*	0.061	0.035*	0.004*	0.036*	Upright visible > upright invisible(5)
	EBA	0.041*	0.014*	0.033*	0.095	0.007*	0.014*	0.036*	Upright visible > upright invisible(6)
	pIPS	0.016*	0.115	0.625	0.176	0.189	0.171	0.458	Upright visible > upright invisible(1)
	mIPS	0.168	0.119	0.955	0.760	0.043*	0.003*	0.646	Upright invisible > upright visible(2)
	aIPS	0.309	0.527	0.765	0.417	0.512	0.470		
	V1	0.189	0.416	0.004*	0.183	0.103	0.751	0.842	Upright visible > upright invisible(1)

The inputs were parameter estimates (percentage signal changes) of GLM per run per condition. Significant results are indicated with *.

The within-participant results in individual participants were consistent with the group results. ANOVA of stream (ventral/dorsal ROIs) × orientation × visibility showed significant interactions of stream × visibility in all seven participants, while the main effect of visibility was present in five participants. The upright visible condition had higher activity than the upright invisible condition in six participants in the combined ventral ROI. In the combined dorsal ROI, however, this comparison was not significant in any of the participants (all *p* > 0.131).

In individual ventral ROIs, a consistent orientation effect was found in both the FBA and the EBA ROIs (six out of seven participants), which was the same case for pairwise comparisons of the upright visible versus invisible bodies.

In individual dorsal ROIs, two participants (S1 and S6) showed higher activity for visible trials, in pIPS and aIPS ROIs. In dorsal ROIs of other participants, the main effect of visibility was either nonsignificant, or showing the opposite effect to ventral ROIs (higher activity for invisible trials than visible ones, in mIPS for one participant), or showing interactions of orientation and visibility (in pIPS for one participant, in mIPS for two participants). One participant further showed a main effect of higher activity for inverted bodies in both pIPS and mIPS, another showed the opposite effect in aIPS. Pairwise comparisons of upright visible and upright invisible conditions showed higher activity for the upright visible condition, in pIPS for one participant, and showed higher activity for the upright invisible condition in mIPS for two participants.

In the V1 ROI, one participant showed the main effect of visibility, showing higher activity for invisible bodies. For pairwise comparisons, another participant showed higher activity for upright visible bodies.

From these results, it appeared that the group-level effects were driven by the majority of the participants, and our results were consistent with the ones found by Fang and He (2005).

## Discussion

Our results show that activity in the dorsal processing stream is relatively independent from visual awareness, strongly contrasting with activity in ventral areas which is strongly linked to the visual awareness. Whole-brain ANOVA showed an interaction effect of stimulus orientation and visibility in regions including the IPS in the dorsal stream, and in subcortical structures. Also, the ROI analysis showed a strong two-way interaction between stream and visibility (validated in within-participant analysis in all seven participants), and a three-way interaction of stream × orientation × visibility, while a main effect of visibility was also present. This overall difference between the two processing streams was caused by different response patterns in posterior and middle IPS ROIs more than the ventral and aIPS ROIs, with the former two areas showing an interaction between stimulus visibility and orientation. Specifically, activity in these two ROIs did not differ between the visible and invisible upright body stimuli. The FBA ROI also showed higher activity for upright bodies than inverted bodies, even when neither was consciously perceived.

The locations of our pIPS and mIPS ROIs correspond to the ROIs of V3A/V7 and IPS in the two previous fMRI CFS studies using tool stimuli (Fang and He, 2005; Hesselmann and Malach, 2011). Our finding that dorsal stream activity for upright body stimuli dissociates from visual awareness is consistent with the findings of Fang and He (2005) using tools. The similarity between our results and theirs underscores that not only tools but also bodies trigger action representation, in which the IPS plays an important role (Culham et al., 2006). The other CFS studies did not find an interaction between stream and visibility but found lower activity for invisible tools in both ventral and dorsal streams (Hesselmann et al., 2011; Hesselmann and Malach, 2011; Ludwig et al., 2015). We also found this main effect of visibility in the dorsal areas, especially for the inverted bodies. However, given the significant interaction between orientation and visibility, our evidence does not support an invariant processing across dorsal and ventral streams.

Previous reviews discussed explanations for the discrepancies between the available studies (Yang et al., 2014; Ludwig and Hesselmann, 2015). One is that the presentation of visible trials was different (presented without dynamic noise in separate runs in Fang and He, 2005; but presented with dynamic noise in the same run in Hesselmann et al., 2011, Hesselmann and Malach, 2011; Ludwig et al., 2015). Experiments of nonconscious tool perception have also been criticized, with the reasoning that the results might be shape-specific and caused by the elongated shape only, rather than other tool-specific properties (Yang et al., 2014), although the elongated invisible tools indeed showed an enhanced decodability (Ludwig and Hesselmann, 2015; Ludwig et al., 2015). However, our results show that these two reasons do not fully account for the previous discrepancies and underlying mechanisms. In our study visible and invisible trials were presented within the same run, always with the dynamic noise present. In addition, the body posture stimuli used in our study have elongated shapes in both upright and inverted forms, but body inversion led to a significant interaction with visibility in posterior and middle IPS, indicating that the underlying mechanisms for nonconscious tool and body perception are not likely to be shape-specific in purely lower-level visual-form aspects, but are more likely linked to higher-level processes associated with these two specific categories, especially their ability to trigger action-related processing.

The discrepancies between those studies may instead be caused by the averaging of activity in dorsal ROIs. Our ROI definition was more fine-grained, and gave the same weight to each dorsal ROI (same spherical ROI size across the three areas). We observed a change of response patterns at the group level along the IPS, where the interaction between orientation and visibility in posterior and middle IPS ROIs was not present in the anterior ROIs, indicating a change of involvement and function across these areas, consistent to the functional heterogeneity found along the IPS in previous research (Freud et al., 2016). Our change of responses was also consistent with the CFS study which specifically examined the decodabilities of faces and tools across two streams (Ludwig et al., 2016). In that study, the authors defined inferior and superior dorsal ROIs, roughly corresponding to a location posterior to our pIPS ROIs, and our mIPS ROI, respectively. They found that the decodability was modulated by the mask contrast in the superior dorsal ROIs, but not so in the inferior dorsal ROIs. Taken together, the response patterns of the main conditions along the IPS are likely to be influenced by the size and location of the ROIs, and by the subsequent averaging of the BOLD responses. In view of the heterogeneity of response patterns along the IPS, further studies with higher functional resolution and fine-grained dorsal ROI definition will help to resolve the discrepancies.

Another possible reason for the discrepancies may be related to the active report of percept with button-press in the three previous studies (Hesselmann et al., 2011; Hesselmann and Malach, 2011; Ludwig et al., 2015). Here, we did not use active reports of visibility for each trial, because active reports under rivalry states induce significantly higher brain activity linked to introspection and action, mainly in frontal areas, but also in superior and inferior parietal areas (Frässle et al., 2014). Since the medial and anterior IPS areas are known to be activated by hand actions such as touching, reaching and grasping (Culham et al., 2006), adding a button response per trial would introduce confounds in perceptual tasks aimed to compare ventral and dorsal activities. Indeed, we can observe the influence of a button press task in our data (Figs. 3, 4), as we see that in the dorsal areas the activity for the catch trials was much higher than the main conditions. Further study explicitly comparing brain activity with/without active reports under CFS would shed more light on its actual influence to the dorsal activity. However, the no-report paradigm also has its limitations. Without explicit requirement of subjective reports like button presses, the participants may still form an implicit “report”; while in cases that a participant indeed consciously perceived a stimulus, the stimulus might either be forgotten, or below the participant’s subjective report criteria, or not even reportable (Tsuchiya et al., 2015). These between-participant variabilities could not all be assessed and accounted for by subjective reports, and would possibly have led to the discrepancies in the literature.

Lastly, the previous CFS studies differed in the length of intertrial/block intervals, and in the data analysis methods. Fang and He (2005) presented 20-s blocks of faces and tools, interleaved with 20-s texture blocks as baseline), and only used the average signal of 8-20 s within each block. Together, by presenting the invisible and visible conditions in different runs, there was no cross talk of signals between conditions, and the BOLD signal dropped to baseline in the texture blocks (shown in their Fig. 3 of objects/scrambled objects experiment; their face/tool experiment was of a similar design). On the other hand, the 3 other studies used another design, with considerably shorter ITIs (1-6.5, 1.5-4.5, and 1-5 s) following the trial-by-trial awareness ratings with button presses, and used the GLM to estimate the BOLD signal changes. Under that specific design, the BOLD signal of the button press in the previous trial was likely to overlap with the upcoming trial despite the jitter of ITI, affecting all conditions. Our slow event-related design included sufficiently long ITIs, which allowed the BOLD response to return to baseline (in our case, the average time for the BOLD response to return to baseline was 8-12 s after the end of dynamic noise presentation; Fig. 3). The next trial started 9-11 TRs after the previous trial onset, and started 1 additional TR later for catch trials with the presence of the response screen (2 s). Thus our design precluded any possible confounds related to effects carried over from preceding trials, and resulted in better estimation for responses of single conditions (Friston et al., 1999).

The time course of the main conditions in Figure 3 seemed to show double peaks, especially in the left pulvinar, with an early peak after trial onset, and a later peak after trial offset. Given the long ITI it is unlikely that this is due to contamination from previous trials. It might be related to the prolonged noise pattern displayed after the target stimulus offset (from 1.5 to 2.5 TR after stimulus onset to remove possible afterimages), during which the two eyes were still under a rivalrous situation, where the noise pattern was rivaling with the blank rectangle instead of the stimulus. This corresponded to the mask-only condition in two previous studies, where two related observations were made. Hesselmann et al. (2011) found that the activity of Mondrian mask-only trials were not significantly different from those of the invisible trials in the ROIs they investigated, although showing a trend to significance in IPS. Ludwig et al. (2015) reconstructed the activity of invisible conditions by subtracting the mask-only activity from the mask-plus-stimulus activity, and found that parametrically modulating the mask contrasts did not show a corresponding difference in the activity in the ventral and dorsal ROIs. Both observations suggest that the activity of invisible conditions under CFS was not modulated in an additive manner relating to the inputs of the two eyes. If the second peak in our data was also induced by the rivaling situation of blank rectangle and the noise pattern, it would question the validity of using the mask-only condition as a baseline. We could not disentangle the mask-only effect from the mask-plus-stimulus effect in our study, but future studies with higher temporal resolution may help understand better the mechanism of CFS.

Since we did not have subjective reports of visibility on a trial-to-trial basis, we used a different way of establishing suppression. We screened participants whose percept of stimuli was well suppressed by CFS, and then verified outside the scanner that their percepts closely follow our experimental manipulation of visibility on a trial-to-trial basis. The strict screening resulted in the relatively low number of participants in the current study, which may not represent the whole population well. To avoid creating differences of processing between the two hemispheres, we balanced the presentation of the noise pattern across the two eyes in both the screening and fMRI experiments. A recent study found that the CFS presentation 3–15 min into one eye would enhance its dominance in a subsequent presentation of binocular rivalry (Kim et al., 2017). Although we used short trials, as our screening experiments are relatively long (0.5–1 h), this may contribute to an understanding of why we did not find a large number of participants whose percept of the stimuli was fully suppressed under CFS. In the current fMRI experiment, there was the possibility that the stimuli occasionally broke the suppression for some participants. If this was the case, the activity for the invisible conditions in the ventral ROIs could be affected. However, this cannot account for the sustained activity we observed in the dorsal ROIs for upright bodies. Thus, our findings are robust in the participants we examined. Future studies may benefit from higher sample size, but may also benefit from experimental designs that are less demanding on the performance of the participants.

Activity in the posterior part of the IPS (pIPS and mIPS ROIs), apart from the possibility that it is linked to the action-perception-related aspect of bodies and tools, may also reflect a more general attentional mechanism triggered by the stimulus. The IPS is part of the dorsal attention network and is known to be activated in multiple tasks. It is involved in the direction of attention, eye movements, and detection of salient events (Corbetta and Shulman, 2011), all of which could have played a role in our experiment. We presented the stimuli in the center of the visual fields, and instructed the participants to always fixate centrally on the fixation cross, thus the voluntary spatial attention of participants was always directed to the center of the rectangles. We could not rule out the possibility that there may be a difference of microsaccades between visible and invisible trials, as a previous CFS study found an increase of gaze directing to the locations of invisible stimuli comparing to contralateral control locations (Rothkirch et al., 2012). Also, activity in the ventral pathway is known to be modulated by attention (Gilbert and Li, 2013), and an attentional modulation of activity was found under CFS as early as V1 despite visibility (Watanabe et al., 2011). However, since the subjective percepts of the invisible trials were the same, the attentional mechanism alone could not explain the higher activity we found for invisible upright vs. inverted bodies in the FBA ROI. Instead, an interplay of dorsal and ventral mechanisms may be present, as recent research suggested for object perception (Freud et al., 2016). In our case, after the information from invisible body stimuli is relayed to both dorsal and ventral pathways, the ventral pathway representation may gain a category-specific processing advantage based on shape and orientation of the upright bodies, and the dorsal pathway representation may gain an advantage relating to action-observation-execution information in the upright bodies. These two in turn may drive the involuntary attention and affect the microsaccades.

The human posterior IPS regions may be homologous to the lateral intraparietal area in monkeys (Culham and Kanwisher, 2001), whose activity is modulated by stimulus salience and behavioral relevance (Corbetta and Shulman, 2002; Baluch and Itti, 2011). In our study the interaction between orientation and visibility is shown in the posterior and middle IPS ROIs as well as in the dorsal attentional network clusters from the whole-brain ANOVA. The interaction found in the posterior part of IPS may reflect a salience competition between the body stimuli and the dynamic noise pattern, caused by binocular disparity. If so, the salience and behavioral relevance of body stimuli may well result from the interplay between ventral and dorsal mechanisms. Given that under CFS salient and behaviorally-relevant stimuli were found to break through suppression faster (Yang et al., 2014), our findings suggest that the posterior part of IPS may act as an important transition stage in mediating stimuli entering into awareness by representing the salience of the stimuli. Our findings add to the link between visual perception and action, and are relevant for understanding the neural basis of perception of affective stimuli outside awareness.

## Synthesis

Reviewing Editor: Christophe Bernard, INSERM & Institut de Neurosciences des Systèmes Decisions are customarily a result of the Reviewing Editor and the peer reviewers coming together and discussing their recommendations until a consensus is reached. When revisions are invited, a fact-based synthesis statement explaining their decision and outlining what is needed to prepare a revision will be listed below. The following reviewer(s) agreed to reveal their identity: Antigona Martinez, Martin Hebart.

Dear authors,

The reviewers and myself agree that, although the concept is not entirely novel, you designed a nice paradigm to address conflicting issues in the current literature.

We have one major concern: the very small number of subjects (N=7) and the related statistical analyses. As is, the study is heavily underpowered (the reported effect sizes are meaningless, with confidence intervals probably ranging from 0 to 1), potentially missing important results, and the N is too small to generalize to the population.

There are two ways to fix this.

Either you perform another experiment with appropriate sample size trying to replicate the original effect (12 participants at the very least given we can't really estimate power based on such a small sample). Including additional data would require different statistics, because it would become a conditional hypothesis (given these subjects show an effect, how likely is it that a larger sample shows an effect), which doesn't work with classical null-hypothesis significance testing. We understand that this would delay the manuscript.

Or, You could show that the effects are not driven by individual subjects, but are present in at least 5/7 subjects. This requires examining the effects in each individual subject and reporting the results accordingly.

Minor

Minor:

Extended data 4-1 is critical bar plot which should be included.

lines 294-297: Please explain what kind of ANOVA was run or what data entered into the ANOVA. I don't quite understand how the degrees of freedom could have come about by an ANOVA within each subject, because (1) wouldn't there be a different statistic for each subject and (2) shouldn't the the error degrees of freedom correspond to the number of scans minus the lost df (including those lost by the autocorrelation)?

Fig 3D uses radiological convention, whereas the remaining figure panels are using neurological convention. I would suggest unifying the description by flipping images in Fig 3D, and perhaps adding labels to all figures denoting whether we are dealing with neurological or radiological views (e.g. adding a letter L).

The paper by Ludwig et al. “Differential modulation of visual object processing in dorsal and ventral stream by stimulus visibility, Cortex, 2016” is relevant and should be cited as their results both in ventral and dorsal regions are consistent with these.

Suggestion: Would the reported results based on subjective visibility generalize to objective visibility conditions? After all, subjective visibility may be related to the response biases of individual subjects, and it may be that the set of subjects just tends to have a very strong bias, rather not reporting visibility. Is there any chance to investigate this in the same pool of subjects?

## References

[B1] Baluch F, Itti L (2011) Mechanisms of top-down attention. Trends Neurosci 34:210–224. 10.1016/j.tins.2011.02.003 21439656

[B2] Brainard DH (1997) The psychophysics toolbox. Spat Vis 10:433–436. 9176952

[B3] Carey DP, Harvey M, Milner AD (1996) Visuomotor sensitivity for shape and orientation in a patient with visual form agnosia. Neuropsychologia 34:329–337. 9148189 10.1016/0028-3932(95)00169-7

[B4] Corbetta M, Shulman GL (2002) Control of goal-directed and stimulus-driven attention in the brain. Nat Rev Neurosci 3:201–215. 10.1038/nrn755 11994752

[B5] Corbetta M, Shulman GL (2011) Spatial neglect and attention networks. Annu Rev Neurosci 34:569–599. 10.1146/annurev-neuro-061010-113731 21692662 PMC3790661

[B6] Culham JC, Kanwisher NG (2001) Neuroimaging of cognitive functions in human parietal cortex. Curr Opin Neurobiol 11:157–163. 11301234 10.1016/s0959-4388(00)00191-4

[B7] Culham JC, Cavina-Pratesi C, Singhal A (2006) The role of parietal cortex in visuomotor control: what have we learned from neuroimaging? Neuropsychologia 44:2668–2684. 10.1016/j.neuropsychologia.2005.11.003 16337974

[B8] de Gelder B, Van den Stock J (2011) The bodily expressive action stimulus test (BEAST). Construction and validation of a stimulus basis for measuring perception of whole body expression of emotions. Front Psychol 2:181. 10.3389/fpsyg.2011.00181 21886632 PMC3152787

[B9] de Gelder B, Tamietto M, Van Boxtel G, Goebel R, Sahraie A, Van den Stock J, Stienen BM, Weiskrantz L, Pegna A (2008) Intact navigation skills after bilateral loss of striate cortex. Curr Biol 18:R1128–R1129. 10.1016/j.cub.2008.11.002 19108766

[B10] Fang F, He S (2005) Cortical responses to invisible objects in the human dorsal and ventral pathways. Nat Neurosci 8:1380–1385. 10.1038/nn1537 16136038

[B11] Frässle S, Sommer J, Jansen A, Naber M, Einhäuser W (2014) Binocular rivalry: frontal activity relates to introspection and action but not to perception. J Neurosci 34:1738–1747. 10.1523/JNEUROSCI.4403-13.2014 24478356 PMC6827584

[B12] Freud E, Plaut DC, Behrmann M (2016) 'What' is happening in the dorsal visual pathway. Trends Cogn Sci 20:773–784. 10.1016/j.tics.2016.08.003 27615805

[B13] Friston KJ, Zarahn E, Josephs O, Henson R, Dale AM (1999) Stochastic designs in event-related fMRI. Neuroimage 10:607–619. 10.1006/nimg.1999.0498 10547338

[B14] Gilaie-Dotan S, Gelbard-Sagiv H, Malach R (2010) Perceptual shape sensitivity to upright and inverted faces is reflected in neuronal adaptation. Neuroimage 50:383–395. 10.1016/j.neuroimage.2009.12.077 20044007 PMC3221039

[B15] Gilbert CD, Li W (2013) Top-down influences on visual processing. Nat Rev Neurosci 14:350–363. 10.1038/nrn3476 23595013 PMC3864796

[B16] Hesselmann G, Malach R (2011) The link between fMRI-BOLD activation and perceptual awareness is “stream-invariant” in the human visual system. Cereb Cortex 21:2829–2837. 10.1093/cercor/bhr085 21515713

[B17] Hesselmann G, Hebart M, Malach R (2011) Differential BOLD activity associated with subjective and objective reports during “blindsight” in normal observers. J Neurosci 31:12936–12944. 10.1523/JNEUROSCI.1556-11.2011 21900572 PMC6623391

[B18] Jiang Y, He S (2006) Cortical responses to invisible faces: dissociating subsystems for facial-information processing. Curr Biol 16:2023–2029. 10.1016/j.cub.2006.08.084 17055981

[B19] Johnson-Frey SH (2004) The neural bases of complex tool use in humans. Trends Cogn Sci 8:71–78. 10.1016/j.tics.2003.12.002 15588811

[B20] Karnath HO, Himmelbach M, Rorden C (2002) The subcortical anatomy of human spatial neglect: putamen, caudate nucleus and pulvinar. Brain 125:350–360. 10.1093/brain/awf032 11844735

[B21] Kim HW, Kim CY, Blake R (2017) Monocular perceptual deprivation from interocular suppression temporarily imbalances ocular dominance. Curr Biol 27:884–889. 10.1016/j.cub.2017.01.063 28262490

[B22] Ludwig K, Hesselmann G (2015) Weighing the evidence for a dorsal processing bias under continuous flash suppression. Conscious Cogn 35:251–259. 10.1016/j.concog.2014.12.010 25649867

[B23] Ludwig K, Sterzer P, Kathmann N, Franz VH, Hesselmann G (2013) Learning to detect but not to grasp suppressed visual stimuli. Neuropsychologia 51:2930–2938. 10.1016/j.neuropsychologia.2013.09.035 24096175

[B24] Ludwig K, Kathmann N, Sterzer P, Hesselmann G (2015) Investigating category- and shape-selective neural processing in ventral and dorsal visual stream under interocular suppression. Hum Brain Mapp 36:137–149. 10.1002/hbm.22618 25270984 PMC6869721

[B25] Ludwig K, Sterzer P, Kathmann N, Hesselmann G (2016) Differential modulation of visual object processing in dorsal and ventral stream by stimulus visibility. Cortex 83:113–123. 10.1016/j.cortex.2016.07.002 27504609

[B26] Lundqvist D, Flykt A, Öhman A (1998) The Karolinska directed emotional faces (KDEF), pp 91-630. CD ROM from Department of Clinical Neuroscience, Psychology Section, Karolinska Institutet.

[B27] Lupyan G, Ward EJ (2013) Language can boost otherwise unseen objects into visual awareness. Proc Natl Acad Sci USA 110:14196–14201. 10.1073/pnas.1303312110 23940323 PMC3761589

[B28] Mastropasqua T, Tse PU, Turatto M (2015) Learning of monocular information facilitates breakthrough to awareness during interocular suppression. Atten Percept Psychophys 77:790–803. 10.3758/s13414-015-0839-z 25720759

[B29] McIntosh RD, McClements KI, Schindler I, Cassidy TP, Birchall D, Milner AD (2004) Avoidance of obstacles in the absence of visual awareness. Proc Biol Sci 271:15–20. 10.1098/rspb.2003.2545 15002766 PMC1691557

[B30] Milner AD (2012) Is visual processing in the dorsal stream accessible to consciousness? Proc Biol Sci 279:2289–2298. 10.1098/rspb.2011.2663 22456882 PMC3350678

[B31] Milner AD, Goodale MA (2006) The visual brain in action. Oxford: Oxford University Press.

[B32] Peelen MV, Downing PE (2007) The neural basis of visual body perception. Nat Rev Neurosci 8:636–648. 10.1038/nrn2195 17643089

[B33] Pelli DG (1997) The VideoToolbox software for visual psychophysics: transforming numbers into movies. Spat Vis 10:437–442. 9176953

[B34] Pinsk MA, Arcaro M, Weiner KS, Kalkus JF, Inati SJ, Gross CG, Kastner S (2009) Neural representations of faces and body parts in macaque and human cortex: a comparative FMRI study. J Neurophysiol 101:2581–2600. 10.1152/jn.91198.2008 19225169 PMC2681436

[B35] Reed CL, Stone VE, Bozova S, Tanaka J (2003) The body-inversion effect. Psychol Sci 14:302–308. 10.1111/1467-9280.14431 12807401

[B36] Rizzolatti G, Sinigaglia C (2010) The functional role of the parieto-frontal mirror circuit: interpretations and misinterpretations. Nat Rev Neurosci 11:264–274. 10.1038/nrn2805 20216547

[B37] Rothkirch M, Stein T, Sekutowicz M, Sterzer P (2012) A direct oculomotor correlate of unconscious visual processing. Curr Biol 22:R514–R515. 10.1016/j.cub.2012.04.046 22789995

[B38] Schurger A (2009) A very inexpensive MRI-compatible method for dichoptic visual stimulation. J Neurosci Methods 177:199–202. 10.1016/j.jneumeth.2008.09.028 18973774

[B39] Stein T, Peelen MV (2015) Content-specific expectations enhance stimulus detectability by increasing perceptual sensitivity. J Exp Psychol Gen 144:1089–1104. 10.1037/xge0000109 26460783

[B40] Stein T, Sterzer P, Peelen MV (2012) Privileged detection of conspecifics: evidence from inversion effects during continuous flash suppression. Cognition 125:64–79. 10.1016/j.cognition.2012.06.005 22776239

[B41] Stienen BM, de Gelder B (2011) Fear detection and visual awareness in perceiving bodily expressions. Emotion 11:1182–1189. 10.1037/a0024032 21707146

[B42] Tsuchiya N, Koch C (2005) Continuous flash suppression reduces negative afterimages. Nat Neurosci 8:1096–1101. 10.1038/nn1500 15995700

[B43] Tsuchiya N, Koch C, Gilroy LA, Blake R (2006) Depth of interocular suppression associated with continuous flash suppression, flash suppression, and binocular rivalry. J Vis 6:1068–1078. 10.1167/6.10.6 17132078

[B44] Tsuchiya N, Wilke M, Frässle S, Lamme VA (2015) No-report paradigms: extracting the true neural correlates of consciousness. Trends Cogn Sci 19:757–770. 10.1016/j.tics.2015.10.002 26585549

[B45] Van den Stock J, Tamietto M, Zhan M, Heinecke A, Hervais-Adelman A, Legrand LB, Pegna AJ, de Gelder B (2014) Neural correlates of body and face perception following bilateral destruction of the primary visual cortices. Front Behav Neurosci 8:30. 10.3389/fnbeh.2014.00030 24592218 PMC3923138

[B46] Watanabe M, Cheng K, Murayama Y, Ueno K, Asamizuya T, Tanaka K, Logothetis N (2011) Attention but not awareness modulates the BOLD signal in the human V1 during binocular suppression. Science 334:829–831. 10.1126/science.1203161 22076381

[B47] Yang E, Brascamp J, Kang MS, Blake R (2014) On the use of continuous flash suppression for the study of visual processing outside of awareness. Front Psychol 5:724. 10.3389/fpsyg.2014.00724 25071685 PMC4093749

[B48] Zhan M, Hortensius R, de Gelder B (2015) The body as a tool for anger awareness—differential effects of angry facial and bodily expressions on suppression from awareness. PLoS One 10:e0139768. 10.1371/journal.pone.0139768 26469878 PMC4607361

